# Midwives’ lived experiences of caring for women with mobility disabilities during pregnancy, labour and puerperium in Eswatini: a qualitative study

**DOI:** 10.1186/s12905-024-03032-z

**Published:** 2024-04-01

**Authors:** Annie M. Temane, Fortunate N. Magagula, Anna G. W. Nolte

**Affiliations:** 1 Health Sciences, University of Johannesburg, Johannesburg, South Africa; 2 Mother and Child Nursing, University of Eswatini, Kwaluseni, Eswatini

**Keywords:** Midwives, Experiences, Maternity care, Women with mobility disabilities, Pregnancy, Labour and the puerperium

## Abstract

**Background:**

Midwives encounter various difficulties while aiming to achieve excellence in providing maternity care to women with mobility disabilities. The study aimed to explore and describe midwives’ experiences of caring for women with mobility disabilities during pregnancy, labour and puerperium in Eswatini.

**Methods:**

A qualitative, exploratory, descriptive, contextual research design with a phenomenological approach was followed. Twelve midwives working in maternal health facilities in the Hhohho and Manzini regions in Eswatini were interviewed. Purposive sampling was used to select midwives to participate in the research. In-depth phenomenological interviews were conducted, and Giorgi’s descriptive phenomenological method was used for data analysis.

**Results:**

Three themes emerged from the data analysis: midwives experienced physical and emotional strain in providing maternity care to women with mobility disabilities, they experienced frustration due to the lack of equipment to meet the needs of women with mobility disabilities, and they faced challenges in providing support and holistic care to women with mobility disabilities during pregnancy, labour and puerperium.

**Conclusions:**

Midwives experienced challenges caring for women with mobility disabilities during pregnancy, labour and the puerperium in Eswatini. There is a need to develop and empower midwives with the knowledge and skill to implement guidelines and enact protocols. Moreover, equipment and infrastructure are required to facilitate support and holistic maternity care for women with mobility disabilities.

## Background

Globally, few studies have focused on midwives’ views of providing maternity care to women with mobility disabilities during pregnancy, labour and the puerperium [1]. In The Disabled World [2], the World Health Organisation (WHO) defines ‘disability’ as an umbrella term covering impairments, activity limitations, and participation restrictions. Furthermore, the WHO defines an ‘impairment’ as a problem in bodily function or structure; an ‘activity limitation’ as a difficulty encountered by an individual in executing a task or action; and ‘participation restriction’ as a problem experienced by an individual in various life situations [2]. In this study, mobility disabilities refer to an impairment in the functioning of the upper and lower extremities as experienced by women during pregnancy, labour and the puerperium.

Midwives, as frontline workers in the delivery of maternity care [3] responsible for the lives of the mother and the baby, are accountable for providing competent and holistic care for women during pregnancy, labour and puerperium. As part of healthcare provision, midwives play an important role in ensuring that every woman, including women with mobility disabilities, receives the best maternity care during pregnancy, labour and puerperium. Moridi et al. [4] state that women with mobility disabilities are entitled to feel safe, respected and well cared for by midwives, who must be sufficiently prepared to care for these women.

According to the Global Population Report, [5] more than one billion people have some form of disability. Eswatini is classified as a middle-income setting in the southern African region, measuring 17 000 square kilometres with a population of 1 093 238. Of the population, 76.2% reside in rural areas (833 472), and 23.8% (259 766) reside in urban areas [6]. The economy is largely agricultural as most industries manufacture agricultural products [7]. Of the Eswatini population, 146 554 (13%) live with disabilities, with most being women (87 258; 16%), 22,871 (14.1%) and 26,270 (14.3%) of them reside in the Hhohho and Manzini regions respectively [8]. 15% (125 545) of people with disabilities live in rural areas, and 85% of the disabled population is unemployed [8], which means most of these individuals are economically disadvantaged. Furthermore, according to the Eswatini Central Statistics Office,^8^ 26.5% of people with disabilities have a mobility (walking) disability, with 63.5% of these being women.

Midwives may encounter difficulties while aiming to achieve excellence in providing maternity care to women with mobility disabilities in what may be challenging circumstances [9]. The WHO [10] claims people with disabilities do not receive the health services they need and are thus likely to find healthcare providers have inadequate skills. Lawler et al. [11] argue that ineffective interactions and poor communication with women needing care, particularly among health professionals engaged in providing maternity services, limit these women’s opportunities to participate in decision-making processes during pregnancy, childbirth, and postpartum care. According to the University of Johannesburg, [12] the midwife, together with the mother, have to engage collaboratively in order to come up with opportunities to promote health while removing any challenges that could impede the achievement thereof.

Walsh-Gallagher et al. [13] postulate that healthcare professionals tend to view women with disabilities as liabilities and regard them as high risk; they often exclude them from the individualised plan of care, which leads to an increase in these women’s fears about their maternity care. These challenges frequently result in health disparities and prevent women with mobility disabilities from receiving optimal maternity care. By exploring midwives’ experiences of this phenomenon, guidelines for support can be developed to extend available knowledge on maternity care for women with mobility disabilities during pregnancy, labour and puerperium.

## Methods

### Study design

The aim of the study was to explore and describe midwives’ experiences of caring for women with mobility disabilities during pregnancy, labour and puerperium in the Hhohho and Manzini regions of Eswatini. A qualitative, [14] exploratory, [15] descriptive, [16] contextual [17] research design with a phenomenological approach [18] was applied for this study to gain insight and understanding of the research phenomenon [19]. The phenomenon under study was midwives’ lived experiences caring for women with mobility disabilities during pregnancy, labour and puerperium. The participants were approached face-to-face to participate in the study. The researchers followed the Consolidated Criteria for Reporting Qualitative Research (COREQ) to report on this qualitative study [20]. 

### Setting

The setting for the study was the Hhohho and Manzini regions of Eswatini. The researcher collected data at the site where participants experienced the phenomenon, as emphasised by Yildiz, [21] within the context in which they were comfortable to be interviewed [22]. This setting included maternal health facilities in hospitals and public health units.

### Population and sampling

The study’s population comprised midwives working in maternal health facilities in hospitals and public health units, that is, one referral hospital and one public health unit in the Hhohho region and two referral hospitals and one public health unit in the Manzini region of Eswatini. Purposive sampling was used to select midwives to participate in the study; [16] 12 midwives from both regions were included. The midwives were between the ages of 35 and 55, and all midwives were black in race and identified as females. The years of experience in the field ranged between 5 and 15 years. The criteria for inclusion were midwives who had provided maternity care to women with mobility disabilities during pregnancy, labour and puerperium for a period of not more than two to three years, willing to participate in the study. The sample size was determined by repetitions of key statements about the research phenomenon during data collection, termed data saturation [23]. None of the participants refused to participate in the study.

Table 1 summarises the participants’ demographic characteristics.

**Table 1:** Participants’ demographic characteristics

Participant	Region	Maternal health care facility	Experience (years)	Age	Gender	Race	Last experience with taking care of a woman with a mobility disability (Months)
01	Manzini	Raleigh Fitkin Memorial Hospital	8	45	Female	Black	24
02	Manzini	Raleigh Fitkin Memorial Hospital	7	40	Female	Black	28
03	Manzini	Raleigh Fitkin Memorial Hospital	15	52	Female	Black	23
04	Manzini	Raleigh Fitkin Memorial Hospital antenatal care unit	5	38	Female	Black	27
05	Hhohho	Mbabane Government Hospital	6	40	Female	Black	13
06	Manzini	Mankayane Government Hospital	5	35	Female	Black	11
07	Manzini	Mankayane Government Hospital	7	40	Female	Black	11
08	Hhohho	Mbabane Government Hospital	3	35	Female	Black	6
09	Hhohho	Mbabane Government Hospital	13	45	Female	Black	6
10	Hhohho	Mbabane Government Public Health Unit	12	55	Female	Black	3
11	Hhohho	Mbabane Government Public Health Unit	14	55	Female	Black	3
12	Hhohho	Mbabane Government Public Health Unit	6	42	Female	Black	2

Source: Adapted from Magagula [24]

### Data collection

In-depth phenomenological, face-to-face, individual interviews were conducted to collect data [17]. The researcher who was a Midwifery lecturer held a Master’s Degree in Maternal and Neonatal science at the time of the study requested approval from the Unit manager to seek permission from the midwives to take part in the study. The midwives were given an information letter which included objectives of the study and the reasons for conducting the study. After recruiting midwives and obtaining their written consent to participate in the study and permission to audio-record the interviews, the researcher set up appointments with them for the interviews, and the data collection process commenced. The central question posed to participants was: *How was it for you to care for a woman with a mobility disability during pregnancy, labour and puerperium?* A pilot of the tool was performed on the first participant who met the inclusion criteria and possessed the same characteristics as those of the study sample. The pre-testing question yielded positive results, the participant responded to the question asked and there was no need to rephrase it or further test it.

The interviews were conducted from March 2019 to July 2019 and lasted 30–45 min. The researcher conducted interviews until the data became redundant and repetitive, reflecting that saturation had been reached, in congruence with Fouché et al. [25] In addition, field notes were recorded in a notebook after each in-depth phenomenological interview. No repeat interviews were held. The researcher ensured bracketing by omitting any perceptions from her past experiences that were likely to influence her interpretation of the research findings.

### Data analysis

Before data analysis commenced, data were organised in computer files after being transcribed and translated into narrative form. Data from each participant were coded and stored in the relevant file and kept in a safe place; only the researcher could access the information. Back-up copies were made of all the data, and the master copies were stored in a safe to which only the researcher had access.

Data collection and analysis occurred concurrently. The researcher was guided by Giorgi et al.’s [26] five-step method of data analysis. This entailed the researcher reading all the transcribed data and the entire ‘naïve description’ provided by the participants during the interviews. The demarcation of ‘meaning units’ within narratives followed. In addition, the researcher marked where meaning shifts occurred and transformed meaning units into descriptive expressions. The researcher laid out the general structure of midwives’ experiences. Moreover, an independent coder was provided with the raw data (after signing a confidentiality agreement) to analyse the findings. The researcher and independent coder analysed the data separately and met for a consensus discussion. Both agreed on all the units of analysis, with an inter-coder reliability of 100%.

### Measures of trustworthiness

The research was informed by Guba and Lincoln’s [27] model in relation to credibility, transferability, dependability and confirmability. For credibility, the researcher ensured prolonged engagement in the field [28], peer debriefing, [29] member checking, and an external auditor was used [25]. The study was also presented at a national conference. Transferability refers to the ability to extend the findings of one’s study to comparable environments or participants, as stated by Pitney et al. [30] The researcher ensured the study’s transferability by providing a richly documented account and in-depth description of all aspects and processes of the study protocol. Data saturation also confirmed transferability [23]. Dependability is evident in a study when other researchers are able to follow the researcher’s decision trail [31]. The researcher ensured dependability by densely describing the research process in congruence with Fouché et al.’s [25] guidelines, so that other researchers can follow similar steps of the same research methodology. Confirmability occurs when the research is judged by the way in which the findings and conclusions achieve their aim and are not the result of the researcher’s prior assumptions and preconceptions [32]. The researcher ensured this by remaining true to the research process through reflexivity and not compromising the research process in any way [28]. In addition, the researcher engaged an independent coder and provided a chain of evidence of the entire research process to enable an audit. Therefore, all forms of collected data, including raw data, reflexive journals, [29] notes and transcriptions, were recorded.

### Ethics

Ethical clearance to conduct this study was obtained from the University of Johannesburg Faculty of Health Sciences Higher Degrees Committee (ref. no. HDC-01-50-2018), University of Johannesburg Faculty of Health Research Ethics Committee (ref. no. REC-01-82-2018), and the Eswatini National Health Research Review Board (ref. no. NHRRB982/2018). The researcher applied and adhered to the four principles to be considered when conducting research: autonomy, beneficence, non-maleficence and justice [33]. Autonomy was adhered to by affording the participants the right to choose to participate in the study and by signing a written informed consent form a week after it was given to them before the interviews commenced. Beneficence was ensured through doing good and doing no harm to participants by prioritising the participants’ interests above those of the researcher, and did not engage in any practice that jeopardised their rights. Non-maleficence was observed by eradicating any possible harmful risks in the study; the researcher ensured the safety of the participants by conducting interviews in a familiar, private environment where they felt free and safe from harm. Furthermore, justice was observed by treating all participants equally regardless of their biographical, social and economic status.

## Results

Three themes and categories emerged from the data analysis. Table 2 summarises the themes and categories of midwives’ lived experiences caring for women with mobility disabilities during pregnancy, labour and puerperium in Eswatini.

**Table 2:** Summary of the themes and categories of midwives’ lived experiences caring for women with mobility disabilities during pregnancy, labour and puerperium

Themes	Categories
1. Physical and emotional efforts required from midwives to provide maternity care to women with mobility disabilities	1.1 Midwives experienced that women with mobility disabilities needed assistance getting onto the bed during labour and delivery 1.2 Midwives experienced challenges in manoeuvring women with mobility disabilities during labour 1.3 Midwives experienced anxiety and the need to exercise patience when caring for women with mobility disabilities
2. Lack of equipment to meet the needs of women with mobility disabilities	2.1 Midwives reported a lack of special beds and infrastructure to meet the needs of women with mobility disabilities
3. Challenges in providing holistic care to women with mobility disabilities during pregnancy, labour and puerperium	3.1 Midwives reported a lack of guidelines and protocols in caring holistically for women with mobility disabilities 3.2 Midwives experienced challenges in allowing significant others to support women with mobility disabilities during labour and delivery

Source: Adapted from Magagula [24]

### Theme 1: physical and emotional efforts required from midwives to provide maternity care to women with mobility disabilities

#### Category 1.1: midwives experienced that woman with mobility disabilities needed assistance getting onto the bed during labour and delivery

According to the participants, caring for women with mobility disabilities weighed heavily on them physically as they were required to assist the women onto delivery beds, which were too high for the women to climb up on their own:

*“The beds are too high, they need to be adjustable…unless you change her to another room, we only have one in the other room…but to be honest she delivered on the same high bed with the help…It’s uncomfortable even with me who is normal, how about someone who has a disability? Getting the woman onto the bed is also uncomfortable for us we end up having pain on our backs.” (M3)*.

*“The challenge is that I couldn’t help her to climb on to the bed, because I needed someone to assist when she came for postnatal care as she was even carrying 3 babies, I didn’t know what to do…I eventually went out and asked for assistance from my colleague…” (M10)*.

*“I believe that the equipment should accommodate the women with disability, however, ours is not accommodative to the women…there are no special delivery beds, specifically designed for them because in my opinion the beds have to be shorter so they can be able to get on to them easily…yes so that they can be able to climb on the beds” (M1)*.

#### Category 1.2: midwives experienced challenges in manoeuvring women with mobility disabilities during labour

Midwives reported it was difficult to perform some procedures while progressing these women during labour and delivery. This situation called for some adjustment and improvisation on their part, and they were unsure if it was the right thing to do.

*“Even though she was a bit uncomfortable and anxious because the leg was just straight and could not bend, I reassured her…She had to remove the artificial leg and remain with the stump. I placed her on the lithotomy position. With the other hand she had to hold on to the ankle of the normal foot, even though it was awkward and difficult to manoeuvre, she managed to deliver the baby.” (M1)*.

*“Luckily for us, she didn’t sustain a tear and we were saved from suturing her cause we foresaw difficulties as how we could have done it as she couldn’t open her thighs well due to the disability…yes I had to get a partner to assist, since she couldn’t even open her thighs. She also couldn’t cooperate possibly because of the pain that is also more reason I asked for my colleague to assist.” (M6)*.

*“…yes…let me make an example, in my case she had a fracture, even if the pelvis was gynaecoid, there were problems of finding the right position for her during delivery, when she had to push the baby out…” (M8)*.

*“The one that I saw did not have one leg. She had come for her postnatal care. We assisted and her on the couch, with my colleague. Since she couldn’t keep her legs open, I asked my colleague to keep one of her legs open whilst I examined her.” (M12)*.

#### Category 1.3: midwives experienced anxiety and the need to exercise patience when caring for women with mobility disabilities

The participants experienced an emotional and psychological burden when caring for women with mobility disabilities. They felt unqualified and foresaw difficulties that triggered anxiety, which led to them not knowing what to do and how to handle these women.

*“It was during labour…the woman was limping the woman she was on crutches. The moment she came into the ward I am a human being I just felt sorry for her kutsi (as to) how is she going to take care of the baby, and the hand was somehow deformed.” (M3)*.

*“At first its emotionally draining as an individual you cause you start sympathising…(other midwife chips in)…yes you even find yourself saying things just because you pity her, and in the process they get hurt.” (M6)*.

*“It came as a shock and it was my first experience, it came as a shock as to how I was going to help her as even my experience was limited in that area.” (M7)*.

*“As I was taking care of her it became necessary for me to put myself into her shoes and to bear with her considering her situation….When you see her for the first time you would pity her yet she is now used to it.” (M1)*.

### Theme 2: lack of equipment to meet the needs of women with mobility disabilities

#### Category 2.1: midwives reported a lack of special beds and infrastructure to meet the needs of women with mobility disabilities

Midwives reported their frustration at the lack of sufficient equipment like special beds and examination tables, tailored for women with mobility disabilities. It was a challenge to provide maternity care for women without this equipment.

*“I believe that the infrastructure and equipment should accommodate the women with mobility disability, however, ours is not accommodative to the women…Usually we don’t have the prenatal ward in the maternity, most women who come in the latent phase have to ambulate, or go to the waiting huts and come back when the labour pains are stronger…There are no special delivery beds, specifically designed for them because in my opinion the beds have to be shorter so they can be able to get on to them easily. We do not even have toilets meant for them.” (M1)*.

*“I was anxious as to how was she going to push how to push cause we do not have the right beds when it was time for pushing I asked for assistance…” (M2)*.

*“The challenge is that I couldn’t help her to climb on to the bed, because I needed someone to assist when she came for postnatal care…the beds need to be adjustable so that they are able to be pushed lower for the mother to move from wheel chair to the bed and we pull the bed up again to examine her.” (M11)*.

### Theme 3: challenges in providing holistic care to women with mobility disabilities during pregnancy, labour, and puerperium

#### Category 3.1: Midwives reported a lack of guidelines and protocols in caring holistically for women with mobility disabilities

Midwives emphasised a lack of guidelines, protocols and knowledge about caring holistically for women with mobility disabilities. This resulted in everyone making their own decisions and doing as they saw fit in caring for these women:

*“I think during antenatal care they (the women with mobility disabilities) need to be prepared for labour cause for others the pain is extraordinary, apart from the pain threshold, they also face self-esteem issues, they are looked down upon…I only saw that she was disabled during assessment cause nothing was recorded on the antenatal care card.” (M2)*.

*“I was not aware of the disability at first, I only discovered when she was pushing…she was admitted and progressed by another midwife, I only attended to her when she was pushing… there was nothing written on the nurse’s notes/ handover notes about her disability.” (M5)*.

*“There is no normal practice for a woman with mobility disability when they come and they are in labour, I usually admit regardless of the stage of labour or dilatation…It is not a protocol, it’s a midwife’s prerogative.” (M1)*.

*“We assess and come up with our own discretion even in terms of admitting them (women with mobility disability). Some midwives will admit them regardless of the stage of labour and disregard the protocol that women who come into labour have to ambulate if they are in the latent phase.” (M8)*.

*“There is one that came the past 3 days she has 3 children now and we just scheduled her for c/section because we know that she has been having c/section since she started. Just from looking at the way she walked, we could tell that she couldn’t deliver normally.” (M9)*.

#### Category 3.2: midwives experienced challenges in allowing significant others to support women with mobility disabilities during labour and delivery

Consequent to the challenges in providing holistic care to women with mobility disabilities, midwives experienced challenges in allowing significant others to support these women during labour and delivery.

*“It can depend on the patients themselves, they should decide and we need to be flexible for it to happen…as you can see our labour room also has the issue of privacy…we would need to restructure cause we have beds for 5 or more women in labour room…and then bringing someone from outside could be tricky” (M6)*.

*“Maybe…not sure though, that they can bring their relatives, but maybe, considering staffing limitation…also the issue of discrimination and privacy, they (the women with disabilities) might feel we discriminate against them because they are disabled we now treat them differently.” (M7)*.

*“Maybe if she can (bring her relative) but that’s not necessary, because I can always ask my colleague to assist, unless there is no one…” (M12)*.

## Discussion

Childbirth is a special experience that requires a personal connection between the midwife and the woman giving birth, characterised by successful communication and respect [34]. However, the themes identified in the study indicated that midwives experienced challenges caring for women with mobility disabilities during pregnancy, labour and puerperium based on their limited capacity and preparedness, and lack of protocols to care for these women. They also reported a lack of supportive equipment for women with mobility disabilities. This posed a challenge for them in attending to these women’s specific needs, and they did not always know how to handle the situation appropriately.

One of the themes centred on midwives’ experiences of the physical and emotional efforts required of them to provide maternity care to women with mobility disabilities. They explained women with mobility disabilities required assistance getting onto the bed during labour and delivery, and more manoeuvring was expected of them (as midwives) as they had to adjust their performance and some procedures. The midwives also reported challenges in providing holistic care to women with mobility disabilities during pregnancy, labour and puerperium. Konig-Bachmann et al. [35] reiterate that caring for women with disabilities requires a level of flexibility, adaptation beyond routine procedures, and demands a high degree of improvisation from healthcare providers to ensure high-quality care. Morrison et al. [36] also found that healthcare providers reported difficulties with equipment when providing healthcare for women with physical disabilities; particularly the beds being too high for them to access. Smeltzer et al. [37] similarly allude to the importance of educating and training clinicians to equip them with knowledge and technical skills to provide more effective care to women with physical disabilities.

The midwives also shared that labour and deliveries were further complicated by some women with mobility disabilities not being able to cooperate due to the pain they experienced; others could not change position due to their disability. In a study by Sonalkar et al., [38] healthcare providers described the gynaecologic examination as challenging to complete as it required patience and the ability to be adaptable to different methods and positioning. Similarly, Konig-Bachmann et al. [35] indicate that in order to provide high-quality care for women with disabilities, healthcare providers need to exercise strong flexibility, adapt beyond routine procedures, and engage in a high degree of improvisation. Byrnes and Hickey [39] concur with this study’s findings and state that due to mobility restrictions, it may be difficult to assess the fundal height and foetal growth in women with physical disabilities.

Some midwives reported their caregiving role was emotionally draining as they felt sorry and pitied the women with mobility disabilities; thus, they needed to show compassion and reassure them. According to Mgwili et al., [40] psychoanalytic thinkers associate pity among staff members upon first contact with a physically disabled person as being instigated by personal feelings, stimulated by the disability. The midwives in this study stated they needed to be more patient and adjust their approach to caring for these women. Tarasoff [41] and Schildberger et al. [42] reiterated that healthcare providers seemed uncomfortable with women’s disability, consequently failing to offer needed support. According to Sonalkar et al., [38] healthcare providers reported there would be less fear and concern about hurting women with disabilities if midwives had increased training. Similarly, Mitra et al. [43] mentioned that healthcare providers had a general lack of confidence in their ability to provide adequate maternity care for women with physical disabilities.

Another theme was midwives’ challenges in providing competent and quality care for women with mobility disabilities due to a lack of equipment, including special beds and examination tables to meet these women’s needs. The examination, labour and delivery beds were too high and could not be adjusted for the women to get on by themselves, or even with the assistance of a midwife. In addition, the midwives reported there was no prenatal ward or waiting huts where they could place these women during the latent phase of labour. The midwives further emphasised there were no special toilets for women with mobility disabilities, which made it hazardous and difficult for them. Mitra et al. [43] concur on the barriers to providing maternity care to women with physical disabilities presented from health professionals’ perspectives. The authors indicated that participants from their study reported inaccessible equipment, including examination tables, as a barrier, making it more difficult and time-consuming to care for women with physical disabilities. In addition, Sonalkar et al. [38] said healthcare providers shared their concern about the lack of adjustable examination tables and transfer equipment, thus presenting a barrier to equitable care for women with disabilities.

Midwives further reported a lack of guidelines and protocols. This resulted in everyone making their own decisions and doing as they saw fit in caring for these women, and, in most instances, not recording the disability at all during antenatal care and admission into labour records. They often only discovered that the woman had a mobility disability at a later stage, when they were in labour. Sonalkar et al. [38] reported that healthcare providers felt frustrated and overwhelmed by the uncertainty of whether they made the correct decisions when caring for women with physical disabilities due to the lack of guidelines forcing them to use their own judgement. Mitra et al. [43] determined that most healthcare providers reported a lack of maternity practice guidelines for women with physical disabilities. Also, healthcare providers highlighted the importance of learning about disabilities and having a better understanding of a condition, particularly if it is likely to be exacerbated during pregnancy [44]. The need to make and read the notes on these women’s antenatal care cards or reports was emphasised.

Due to the lack of clear guidelines and protocols in caring for women with mobility disabilities, the midwives reported they sometimes admitted the woman into the labour ward regardless of the stage of labour, while other midwives did not and wanted them to walk around and come back for admission once they are in the active phase of labour. Furthermore, the midwives explained they often referred these women for caesarean sections right away, regardless of whether the woman could deliver normally due to mere panic from just seeing the disability or based on a previous record of surgery. Smeltzer et al. [45] researched obstetric clinicians’ experiences and educational preparation in caring for pregnant women with physical disabilities, and they agree on the lack of knowledge among health professionals caring for women with mobility disability.

Devkota et al. [46] also agree regarding midwives’ inefficiency in providing quality care for women with mobility disabilities. They claim healthcare providers often struggle to understand women with disabilities’ needs as they are not formally trained to provide services to this population. These healthcare providers were found to be undertrained in specific skills that would equip them to provide better and more targeted services for women with disabilities.

Consequent to the challenges in providing holistic care to women with mobility disabilities during pregnancy, labour and puerperium, midwives experienced challenges in allowing significant others to support these women. They reported that as much as they needed assistance caring for these women, and as much as the women would prefer to have their family members or significant others assisting them, this is not possible due to the lack of privacy, especially in public health facilities. Walsh-Gallager et al.’s [13] study on the ambiguity of disabled women’s experiences of pregnancy, childbirth and motherhood resonate with this study’s findings. The authors reported that women with disabilities’ partners were denied access or had their visits curtailed on several occasions due to inflexible hospital visiting policies. Redshaw et al. [47] reiterated the same in their study; disabled women were less likely to say their companion or partner was welcome to visit, let alone provide any form of assistance. In addition, a study by Bassoumah and Mohammad [48] reported that women with disabilities were denied their spouses’ support while receiving maternity care. Byrnes and Hickey [39] also concur that every effort should be made to allow women with disabilities who are in labour to receive support from significant others, and they should be active partners in the labour process.

### Limitations

The study was limited to two of the four regions of Eswatini, namely Hhohho and Manzini; hence, the results could not be generalised for the whole country. The study also only focused on mobility disabilities due to time constraints and limited funds. Future research could be conducted to cover all other forms of disabilities.

## Conclusion

This study focused on midwives’ lived experiences caring for women with mobility disabilities during pregnancy, labour and puerperium in Eswatini. In-depth phenomenological interviews were conducted, the findings were analysed, and themes were established. The findings illustrate that midwives experienced challenges caring for women with mobility disabilities during pregnancy, labour and puerperium in Eswatini. There is a need to develop and implement guidelines to empower midwives with knowledge and skill to provide support and holistic maternity care, and enact protocols. They should also have access to appropriate equipment and infrastructure specifically tailored towards promoting optimal health for women with mobility disabilities.

## Data Availability

The data analysed is available from the corresponding author upon reasonable request.
